# Modulation Doping Enables Ultrahigh Power Factor and Thermoelectric ZT in n‐Type Bi_2_Te_2.7_Se_0.3_

**DOI:** 10.1002/advs.202201353

**Published:** 2022-04-27

**Authors:** Cheng‐Lung Chen, Te‐Hsien Wang, Zih‐Gin Yu, Yohanes Hutabalian, Ranganayakulu K. Vankayala, Chao‐Chih Chen, Wen‐Pin Hsieh, Horng‐Tay Jeng, Da‐Hua Wei, Yang‐Yuan Chen

**Affiliations:** ^1^ Institute of Physics Academia Sinica Taipei Taiwan 11529 ROC; ^2^ Department of Physics National Chung Hsing University Taichung Taiwan 40227 ROC; ^3^ Graduate Institute of Manufacturing Technology National Taipei University of Technology Taipei Taiwan 10608 ROC; ^4^ Institute of Earth Sciences Academia Sinica Taipei Taiwan 11529 ROC; ^5^ Department of Physics National Tsing Hua University Hsinchu Taiwan 30013 ROC; ^6^ Graduate Institute of Applied Physics National Chengchi University Taipei Taiwan 11605 ROC

**Keywords:** Bi_2_Te_3_, energy generation, intercalation, modulation doping, thermoelectric materials

## Abstract

Bismuth telluride‐based thermoelectric (TE) materials are historically recognized as the best p‐type (ZT = 1.8) TE materials at room temperature. However, the poor performance of n‐type (ZT≈1.0) counterparts seriously reduces the efficiency of the device. Such performance imbalance severely impedes its TE applications either in electrical generation or refrigeration. Here, a strategy to boost n‐type Bi_2_Te_2.7_Se_0.3_ crystals up to ZT = 1.42 near room temperature by a two‐stage process is reported, that is, step 1: stabilizing Seebeck coefficient by CuI doping; step 2: boosting power factor (PF) by synergistically optimizing phonon and carrier transport via thermal‐driven Cu intercalation in the van der Waals (vdW) gaps. Theoretical ab initio calculations disclose that these intercalated Cu atoms act as modulation doping and contribute conduction electrons of wavefunction spatially separated from the Cu atoms themselves, which simultaneously lead to large carrier concentration and high mobility. As a result, an ultra‐high PF ≈63.5 µW cm^−1^ K^−2^ at 300 K and a highest average ZT = 1.36 at 300–450 K are realized, which outperform all n‐type bismuth telluride materials ever reported. The work offers a new approach to improving n‐type layered TE materials.

## Graphical abstract

The intercalated Cu atoms in the Bi_2_Te_2.7_Se_0.3_ structure act as modulation doping and effectively improve carrier mobility while maintaining the carrier concentration similar to that in the uniformly doped sample. A record‐high ZT of 1.42 at 375 K is successfully realized in (CuI)_0.002_Bi_2_Te_2.7_Se_0.3_ + 0.2 % Cu.

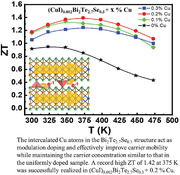

## 1 Introduction

Thermoelectric (TE) material is a kind of material that can directly convert waste heat into useful electricity or be used for cooling applications. Because of the advantage of scalability, noise‐free operation, simplicity, high reliability, and environmental friendliness, TE energy technology has recently attracted global interest in the field of power generation.^[^ ^1^, ^2^, ^3^ ^]^ The TE performance of materials is usually evaluated by the dimensionless figure‐of‐merit, ZT = *S^2^σ*T/*κ*, where *S* is the Seebeck coefficient, *σ* is the electrical conductivity, the product *S^2^σ* is the power factor (PF), *κ* is the thermal conductivity, and T is the absolute temperature, respectively.^[^ ^4^ ^]^ A promising TE material should have a high PF and a low thermal conductivity.

Despite that a great variety of novel TE materials have been proposed and thoroughly studied, bismuth telluride (Bi_2_Te_3_)‐based alloys are still the most promising material for industrial applications near room temperature. Bi_x_Sb_2‐x_Te_3_ and Bi_2_Te_3‐x_Se_x_ (BTS) are the typical p‐type and n‐type materials of these alloys, respectively. The maximum ZT of p‐type materials has recently been boosted to as high as 1.8 by introducing sophisticated nanostructures;^[^ ^5^ ^]^ nevertheless, few similar breakthroughs have occurred in their n‐type counterparts. Such a serious TE performance imbalance between p‐ and n‐type Bi_2_Te_3_‐based materials severely blocks the realization of energy‐efficient TE devices. Extensive endeavor has been undertaken to improve TE performance of n‐type BTS materials via textured microstructures and point defect engineering.^[^ ^6^, ^7^, ^8^, ^9^, ^10^ ^]^ A promising example is the use of liquid tellurium phase sintering to effectively scatter phonons of all frequencies and thereby obtain a high ZT≈1.4 at 425 K.^[^ ^7^ ^]^ However, the primary gain in ZT comes from a low thermal conductivity, with little improvement in PF, which greatly limits the scope for further improvement in overall performance.

Bi_2_Te_3_ alloys crystallize in a rhombohedral structure, consisting of three quintuple layers along the *c*‐axis, and show a strong anisotropic nature in their thermal and electrical transport properties.^[^ ^11^ ^]^ Their electrical and thermal conductivities along the basal plane were reported to be about ≈2–4 times larger than those along the *c*‐axis.^[^ ^12^ ^]^ In contrast, the Seebeck coefficient exhibits a relatively weak anisotropic behavior. As a result, the best ZT value is always expected along the basal plane, especially for n‐type BTS alloys, which show a more significant anisotropy in the electrical conductivity than the thermal conductivity. Therefore, the degree of structure texturing is crucial in enhancing the PF and even ZT for n‐type BTS alloys.^[^ ^13^, ^14^ ^]^

In n‐type Bi_2_(Te,Se)_3_ based alloys, the number of vacancies or antisite defects generated in the manufacturing process is also another major factor that drastically affects their TE properties. For example, Bi vacancies (*V*_Bi_) and anion vacancies (*V*_Te_ or *V*_Se_) are easily formed during the mechanical deformation treatments while the anti‐site defects (Bi_Te_ or Bi_Se_) are inherently generated during the melt‐grown process.^[^ ^15^ ^]^ Generally, these excess anion vacancies are called donor‐like defects, which mainly change the point defect concentration of n‐type BTS and thus affect the carrier concentration and the Seebeck coefficient. To weaken the donor‐like defects, halogen atoms like Br or I are often introduced to occupy anion vacancies (*V*_Te_ or *V*_Se_), thereby smoothly optimizing the carrier concentration.^[^ ^16^ ^]^

**Figure 1:**
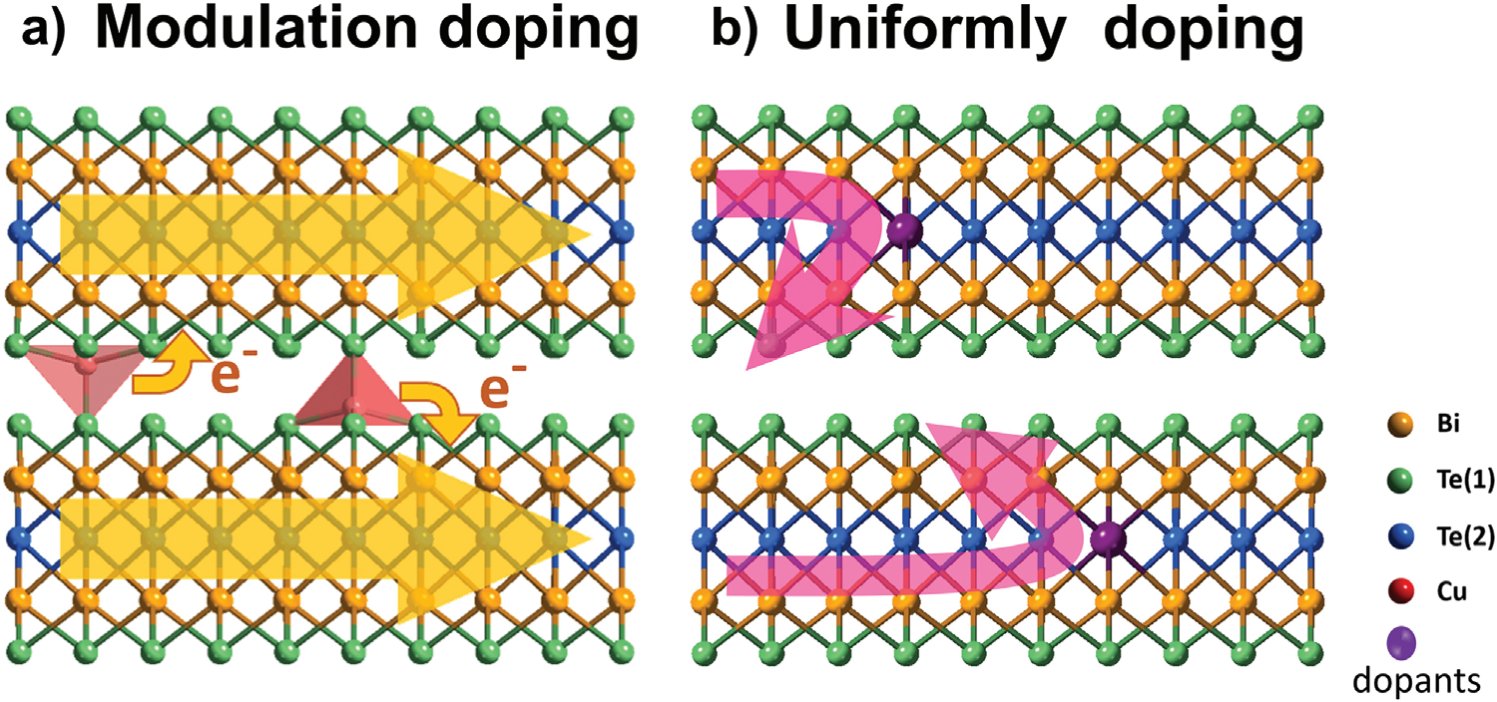
Schematic diagrams of modulation doping and uniformly doping in Bi_2_Te_3_. For modulation doping, the carriers are spatially separated from their intercalated atoms, which shows higher carrier mobility than that of the uniformly doping.

Modulation doping is a well‐developed technique in microelectronics, photonic components, and even 2D materials to increase carrier mobility and thus electrical conductivity.^[^ ^17^ ^]^ The key mechanism of modulation doping is that the doped layer provides carriers and the carriers are transported in the undoped layer, this mechanism can reduce the impurity scattering and enhance the mobility of carriers. MgZnO/ZnO heterostructures, BiCuSeO systems, Bi_2_O_2_Se layered semiconductors, and Si_1‐x_Ge_x_ composites are typical examples.^[^ ^18^, ^19^, ^20^ ^]^ Especially, ultrahigh carrier mobility of 180 000 cm^2^ V^−1^ s^−1^ was obtained in the MgZnO/ZnO heterostructure grown by molecular beam epitaxy, which is about 9 times larger than that of the defect‐free single‐crystal ZnO crystal. Modulation doping mechanisms are mostly limited to 2D materials, and there are few studies on the 3D bulk structure materials and the TE materials. Recently, modulation doping was realized in Si_1‐x_Ge_x_ heterostructure consisting of a periodic doped and undoped blocks.^[^ ^20^ ^]^ Modulation doping on these TE materials did enhance the PF and ZT by mobility enhancement. These reports inspired us to apply modulation doping in an n‐type BTS system with 2D layered structure (**Figure** **1**). In this work, the significant improvement in carrier mobility/PF, and the suppression of phonon heat transport were accomplished in the n‐type Bi_2_Te_2.7_Se_0.3_ crystals showing a record high ZT = 1.42 at room temperature.

Recently, Cu atoms or Cu(I)‐halide adducts are proposed as promising additives to realize a high ZT in n‐type BTS alloys.^[^ ^21^, ^22^, ^23^, ^24^, ^25^ ^]^ Cu is an amphoteric dopant. It can act as an acceptor or a donor depending on its location in the BTS lattice.^[^ ^26^ ^]^ Several groups have demonstrated that moderate Cu intercalation in BTS not only changes the electronic properties but also suppresses the thermal conductivity, thereby improving the overall TE performance.^[^ ^27^, ^28^, ^29^, ^30^ ^]^ However, it is worth noting that excessive doping and/ or improper placement of Cu will easily form Cu‐rich nanoprecipitates in the matrix and lead to carrier mobility degradation in parent materials. The maximum ZT in their studies is around ≈0.9–1.2, and seems to reach the limit. In these studies, several issues such as how many Cu atoms are intercalated in the vdW gap, how to precisely control the number of intercalated Cu atoms, and how much impact it has on the TE properties, have not yet been thoroughly discussed. The intercalation of the materials is the process of inserting foreign atoms/ ions into the vdW gaps of the layered materials. It has recently been proved to be a powerful avenue to control the physical properties of 2D layered materials.^[^ ^31^, ^32^, ^33^ ^]^ Several advantages are as follows: 1) intercalation can make the doping concentration break through the limits of general metallurgical technology; 2) the intercalation process is controllable by spontaneous self‐intercalation or electrochemical method; 3) in situ real‐time observing the property changes during the intercalation process; 4) intercalation can lead to structural changes, such as lattice expansion or even phase change. Combining the above characteristics, the intercalation technique indeed provides a new degree of freedom to tune layered materials, and has great potential to be applied to enhance the TE performance of Bi_2_Te_3_‐based materials.

Herein, we report a new strategy to make it easier to control the intercalation of Cu atoms into the vdW gap of n‐type Bi_2_Te_2.7_Se_0.3_ crystals via a thermal‐driven approach, and investigate the corresponding TE properties. Particularly, we found that the intercalated Cu atoms can not only increase the carrier concentration but also maintain high mobility, thus significantly enhancing the PF of the material to an ultrahigh value ≈63.5 µW cm^−1^ K^−2^ at 300 K. Moreover, the Cu atoms intercalated in the vdW gap play an important role in scattering phonons, resulting in a considerable reduction in lattice thermal conductivity. As a result, a high peak ZT of 1.42 at 375 K and a high average ZT of 1.36 in the temperature range of 300–450 K were achieved for (CuI)_0.002_Bi_2_Te_2.7_Se_0.3_ + 0.2% intercalated Cu sample.

## 2 Result and Discussion

In our pre‐experimental measurements, we found that (CuI)_0.002_Bi_2_Te_2.7_Se_0.3_ + *y* % Cu (*y* = 0.1, 0.2, and 0.3) crystals exhibited the best TE properties along basal plan as that of Bi_2_Te_3_‐based materials, so all following TE properties in this work were performed along the basal plane in order to obtain the best TE transport performance. The pristine Bi_2_Te_2.7_Se_0.3_ shows a positive Seebeck coefficient (*p*‐type) due to the Bi_Te_ anti‐site defects. When adding CuI dopants into the Bi_2_Te_2.7_Se_0.3_, the substantial electron donors contributed by Cu‐ and I‐doping change the Seebeck coefficient to a negative value (*n*‐type). An appropriate CuI doping is crucial to maximizing ZT by optimizing the thermal and electrical transport properties. The (CuI)_x_Bi_2_Te_2.7_Se_0.3_ (x = 0.002) crystal with the highest ZT of 1.0 at 350 K was chosen as the base material to explore the novel TE properties after Cu intercalation. The detailed structural characterization and TE properties of (CuI)_x_Bi_2_Te_2.7_Se_0.3_ (x = ≈0–0.004) crystals are presented in Figures S1–S3, Supporting Information.

### 2.1 Thermal‐Driven Cu Intercalation in (CuI)_0.002_Bi_2_Te_2.7_Se_0.3_

Although CuI doping is helpful in improving the TE properties of n‐type Bi_2_Te_2.7_Se_0.3_ materials, the ZT enhancement of the materials is limited to a maximum of 1.0 with optimal CuI doping, that is, x = 0.002. The reason is that doping with more CuI by high‐temperature melting method did not make the added copper intercalated in the vdWs gap, but will form more Cu or CuI nano‐precipitations.^[^ ^30^ ^]^ These nano‐precipitates have the chance to increase phonon scattering and are very unfavorable to carrier transport. In this study, we found that copper atoms can be effectively intercalated into the vdWs gap by thermal diffusion, and play an important role in regulating the TE properties of the material. The (CuI)_0.002_Bi_2_Te_2.7_Se_0.3_ crystal with high ZT was therefore chosen as the base material to explore the novel TE properties for further Cu intercalation.

**Figure 2:**
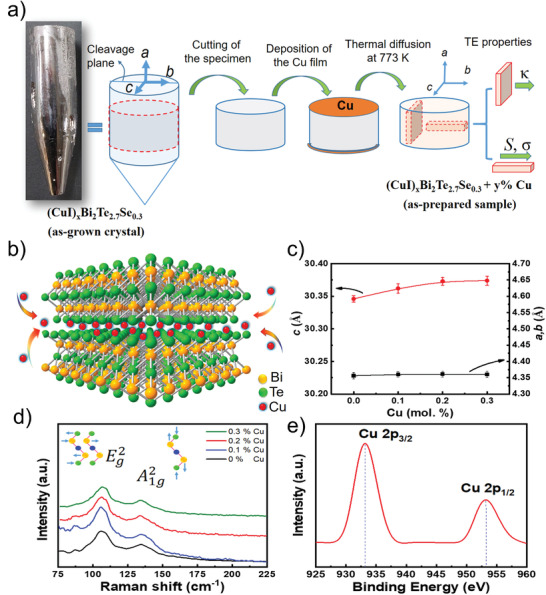
Preparations and Characterizations of (CuI)_0.002_Bi_2_Te_2.7_Se_0.3_ + *y* % Cu crystals. a) The schematic diagram of the preparation process and transport measurements of Cu‐intercalated samples. b) Illustration of the Bi_2_Te_3_ structure with Cu atoms intercalated into the vdWs gap. c) Variation of the lattice constants. d) Comparison of the Raman spectra for all samples. e) The selected XPS spectra nearby the Cu 2*p* orbitals for (CuI)_0.002_Bi_2_Te_2.7_Se_0.3_ + 0.2% Cu intercalants, presenting only two Cu zero‐valence characteristic peaks at 933 and 953 eV for Cu 2*p*_3_ *_/_*_2_ and Cu 2*p*_1_ *_/_*_2_, respectively.

The number of intercalated Cu atoms is calculated based on the number of Cu atoms per unit volume. Taking the sample with 0.2% Cu intercalation as an example, it is approximately equivalent to 1.5×10^19^ Cu atoms cm^−3^. According to the volume size of the crystal, we estimate how much thickness (equivalent weight) of Cu needs to be thermally deposited on the crystal surface. However, the layered structural properties of Bi_2_Te_3_ result in anisotropic diffusion of Cu atoms within the structure.^[^ ^34^ ^]^ It is better to deposit Cu on the surface that is parallel to the c‐axis of the crystal, which make Cu diffuse smoothly into vdWs gaps via thermal‐driven approach (**Figure** **2**a). Moreover, at sufficient temperature and time, Cu atoms can diffuse across the terraces and uniformly distribute throughout the crystal. Experimentally, the sample with evaporated Cu film will be heat treated at 773 K for several days and then quenched to room temperature. The annealing time depends on the sample size. For example, a sample with a diameter of 10 mm and a height of 13 mm will take about 3–4 days. It should be noted that the quenching process is critical to the homogeneity of Cu atoms in this thermal‐driven intercalation approach.

Figure 2b illustrates the crystal structure of Bi_2_Te_3_. Each quintuple layer is composed of five covalently bonded atomic planes Te—Bi—Te—Bi—Te, and they are combined through vdWs interaction to form Bi_2_Te_3_ crystals. It is proposed that the thermally diffused Cu atoms can be readily intercalated and located between the quintuple layers. Figure 2c shows the lattice constants as a function of Cu content in (CuI)_0.002_Bi_2_Te_2.7_Se_0.3_ compounds. The lattice constant increases from 30.341 to 30.375Å in *c*‐axis whereas almost constant in *a* and *b* axes with increasing Cu content from *y* = 0 to 0.3 mol. %. The increased *c*‐axis value is attributed to the fact that Cu atoms enter the interstitial positions formed by four‐Te(1) atoms and increase the distance between quintuple layers.^[^ ^27^ ^]^ The above results can also be subtly shown from the Raman spectrum, which shows a little shift in the out‐of‐plane lattice vibration of $A_{1g}^{2}$ mode (134.7 cm^−1^ with Cu and 136.0 cm^−1^ without Cu, Figure 2d). It is noted that the two prominent peaks at 103 and 136 cm^−1^ are assigned to $E_{g}^{2}$ and $A_{1g}^{2}$ modes of (CuI)_0.002_Bi_2_Te_2.7_Se_0.3_, respectively. The $A_{1g}^{2}$ mode corresponds to the vibration mode along the *c*‐axis; the$\;E_{g}^{2}$ mode corresponds to the in‐plane vibrations that are perpendicular to the c‐axis. This is consistent with the prior observation for Bi_2_Se_3_ nanoribbons with Cu intercalation.^[^ ^35^ ^]^ Furthermore, the two characteristic peaks of XPS (953 and 933 eV) for zero‐valence Cu are observed in this intercalation system, which further proves that the zero‐valence Cu is intercalated within the vdWs gap instead of Cu ions (Figure 2e).

**Figure 3:**
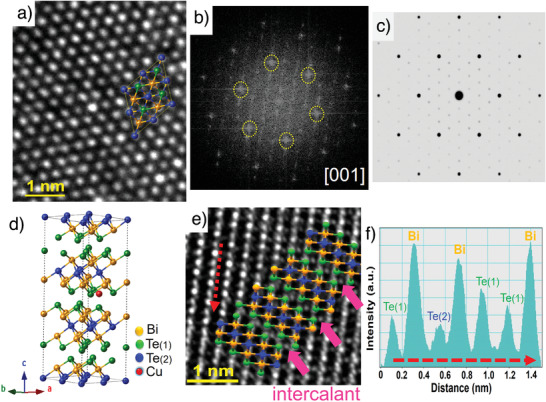
Microstructures of (CuI)_0.002_Bi_2_Te_2.7_Se_0.3_ subjected to 0.2% Cu intercalation. a) HRTEM images viewed along *c*‐axis direction with the corresponding b) FFT analysis. c) A simulated electron diffraction pattern based on the model of d) Cu intercalated within Bi_2_Te_3_ structure. e) The Cs‐HRTEM image of the specimen observed along [100] crystallography zone axis. f) Intensity line profile analyzed along the red color arrow shown in (e) reveals the arrangement of Bi and Te in the lattice.

To understand and ensure that the introduced Cu atoms intercalated into the vdWs gap, we investigated the microstructures of 0.2% Cu intercalated specimen using a spherical aberration‐corrected high‐resolution transmission electron microscope (Cs‐HRTEM). A hexagonal lattice was observed along *c*‐axis direction in the high‐resolution images (**Figure** **3**a), and the atomic positions of Bi and Te(Se) are outlined by the overlaid Bi_2_Te_3_ structural model. The structural morphology is not altered with intercalation, and no precipitates are observed on the surface of the intercalated specimen. Figure 3b shows the corresponding fast Fourier transform (FFT) analysis conducted from Figure 3a, and presents obvious satellite spots associated with each Bi_2_Te_3_ Bragg spot (dashed yellow circles). This result is consistent with the Bi_2_Te_3_ diffraction spots (big black spots) and superlattice diffraction spots (small black spots) simulated based on the model of Cu intercalated in the vdWs gap of Bi_2_Te_3_ structure (Figure 3c,d). Similar satellite point features are generally recognized to exist in the superlattice intercalation structure.^[^ ^36^ ^]^ Furthermore, we also acquired a Cs‐HRTEM image of the same specimen along [100] crystallography zone axis to more clearly distinguish the distributions of atoms, as shown in Figure 3e. The position of Bi and Te atoms were identified by intensity line profile and intercalated Cu atoms were represented by small grey dots as marked by the pink arrows in the image, demonstrating the exact position of Cu atoms. Figure 3f illustrates the intensity profile of the image in Figure 3e, and points out the positions of Bi and Te in the lattice arrangement according to the difference in their atomic numbers. In contrast with the pristine Bi_2_Te_2.7_Se_0.3_, no such result was ever observed. Therefore, it is reasonable to conclude that most intercalated Cu atoms are indeed placed at the vdWs gap. Furthermore, the compositions and the distributions of Cu, Bi, Te, and Se elements in (CuI)_0.002_Bi_2_Te_2.7_Se_0.3_ + 0.2% Cu sample were analyzed using an electron probe microanalyzer, confirming the homogeneous distribution of elements in the specimen (Figure S4, Supporting Information).

### 2.2 Role of Cu Intercalation in Enhancement of Power Factor

**Figure 4:**
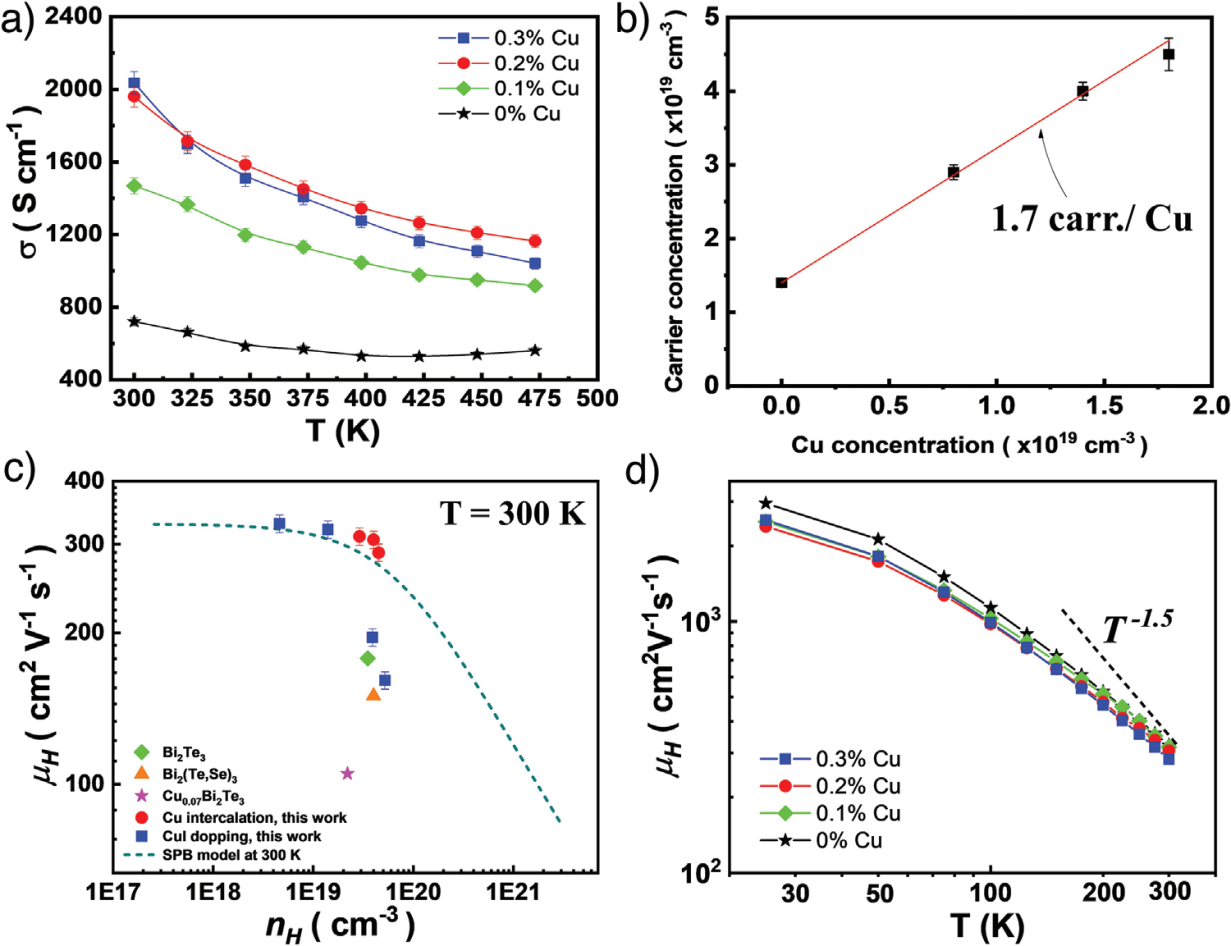
Electrical transport properties of (CuI)_0.002_Bi_2_Te_2.7_Se_0.3_ + *y* % Cu crystals. a) Electrical conductivity. b) Hall carrier concentration as a function of intercalated Cu concentration. c) Hall mobility as a function of carrier concentration at 300 K. The reported data from representative single‐crystal Bi_2_Te_3_,^[^ ^38^ ^]^ Cu_0.07_Bi_2_Te_3_,^[^ ^39^ ^]^ and Bi_2_(Te,Se)_3_^[^ ^40^ ^]^ are also given for comparison. d) Temperature dependence of carrier mobility *μ_H_*.

The temperature dependence of electrical conductivity (*σ*) of (CuI)_0.002_Bi_2_Te_2.7_Se_0.3_ + *y* % Cu crystals enhances notably with the higher mole fraction of Cu intercalation (**Figure** **4**a). For example, the *σ* rises from ≈721 S cm^−1^ for 0% Cu to ≈2036 S cm^−1^ for 0.3% Cu intercalation at 300 K. According to the Hall measurement results, we can clearly clarify that as *n*_H_ increases with the number of intercalated Cu atoms, *μ* is only slightly affected, so the *σ* is greatly enhanced (**Table** **1**). We further evaluate the number of free electrons provided by each intercalated Cu by analyzing the relationship between Hall carrier concentration and the concentration of intercalated Cu atoms (Figure 4b). We found that each intercalated Cu is capable to contribute about 1.7 electrons to the crystal. The value is quite comparable to 1.4 electrons that CuI doping can offer (see Figure 4b for y = 0). In fact, Cu atoms could play different roles in different situations depending on the experimental process of preparing the specimens. For example, it was reported that the substitution of Bi with Cu can act as p‐type doping decreasing the electronic concentration of n‐type Bi_2_Te_3_.^[^ ^37^ ^]^ Generally, the Cu atoms would tend to act as donors because of its 4s^1^d^10^ electron configuration. The Cu would like to give out an electron to achieve a stable closed‐shell electron configuration. This is more likely true for Cu intercalation in the vdWs gap because the intercalated Cu is lacking strong bonding with other atoms so the Cu atom would prefer to keep its original atomic property, that is, tend to donate its s electron. To verify this, we calculate the charge transfer analysis (CTA) of Cu‐intercalated Bi_2_Te_3_ (Figure S5, Supporting Information). The CTA shows the difference in the valence charge density between the real Cu‐doped Bi_2_Te_3_ and that in which each atom keeps its charge density the same as an isolated atom. As can be seen, the CTA indeed shows strong negative values around the intercalated Cu indicating it indeed acts as a donor.

**Table 1:** The Hall carrier concentration (*n*_H_), mobility (*μ*), electrical conductivity (*σ*), Seebeck coefficient (*S*), and PF of (CuI)_0.002_Bi_2_Te_2.7_Se_0.3_ + *y* % Cu crystals at 300 K

Transport properties of (CuI)_0.002_Bi_2_Te_2.7_Se_0.3_ + y% Cu					
Samples	*σ* [S cm^−1^]	*n*_H_ [×10^19^ cm^−3^]	*μ* [cm^2^ V^−1^ s^−1^]	*S* [µV K^−1^]	PF [µWcm^−1^ K^−2^]
(CuI)_0.002_Bi_2_Te_2.7_Se_0.3_	721	1.4	321	−251	45.4
(CuI)_0.002_Bi_2_Te_2.7_Se_0.3_ + 0.1% Cu	1468	2.9	311	−202	59.9
(CuI)_0.002_Bi_2_Te_2.7_Se_0.3_ + 0.2% Cu	1961	4.0	306	−180	63.5
(CuI)_0.002_BiTe_2.7_Se_0.3_ +0.3% Cu	2036	4.5	288	−170	58.8

More importantly, the intercalation of Cu atoms in Bi_2_Te_2.7_Se_0.3_ crystals hardly affects the carrier mobility but can effectively tune the carrier concentration to a similar level as CuI doping can achieve (Figure 4c). The carrier mobility of modulation‐doped (CuI)_0.002_Bi_2_Te_2.7_Se_0.3_ + 0.2% Cu sample is about 306 cm^2^ V^−1^ s^−1^, which is almost 2 to 3 times higher than that of other heavily doped single crystals with similar carrier concentrations (4.0×10^19^ cm^−3^).^[^ ^38^, ^39^, ^40^ ^]^ The significant reduction in carrier mobility after typical doping indicates a strong scattering of carriers. This amazing result verifies the unique role of Cu intercalation in regulating charge transport in *n*‐type Bi_2_Te_2.7_Se_0.3_ materials. For all samples, the temperature dependence of Hall mobility decreases with temperature roughly following a T^−1.5^ relation (Figure 4d). This result allows us to use the simple single parabolic band (SPB) model with acoustic phonon scattering assumption to clarify the change of transport properties with Cu intercalation.

**Figure 5:**
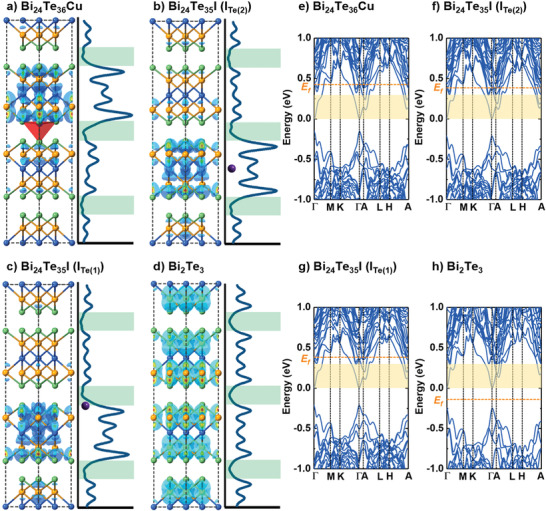
Electronic structure and charge density of conduction electrons for Cu‐doped and I‐doped Bi_2_Te_3_. (a,b) Average charge density of the states over the energy 0–0.3 eV above the CBM indicated by the orange shaded region in (e–h). Its average over the plane normal to the *c* direction is plotted at the right side of each panel. The green shaded regions denote the vdWs gap. In (b,c), the position of the iodine substituting the Te(1)/Te(2) is denoted, for clarity, by the purple dot. (e–h) Electronic structure, in which the energy zero is set at CBM, and the Fermi level, *E_f_*, is indicated by the orange dashed line.

To gain physical insight into our measured high mobility, we calculate the electronic structures of Cu‐doped, I‐doped, and undoped Bi_2_Te_3_. The doped Cu atoms have been demonstrated preferentially to intercalate into the vdWs gap on the tetrahedral site between the Te(1) layers (**Figure** **5**a), and the doped I atoms tend to substitute Te atoms (Figure 5b,c).^[^ ^35^, ^41^ ^]^ Both the Te(1) and Te(2) atoms could be substituted by I atoms because of the comparable formation energies.^[^ ^41^ ^]^ As shown in Figure 5e–h, both I and Cu atoms act as donors, raising the Fermi level from the energy gap (Figure 5h) to the energy above the conduction band minimum (CBM) in agreement with the results of Hall measurements.

For n‐type doping, the electrical conductivity is mostly determined by the conduction electrons around CBM, thus, around which, we calculate the average charge density over the electronic states as plotted in Figure 5a–d. As can be seen, the charge density of the conduction electrons inside the quintuple layer closest to the intercalated Cu (Figure 5a) and that inside the quintuple layer whose Te atom is substituted by I (Figure 5b,c) are considerably larger than that of the pure Bi_2_Te_3_ (Figure 5d). In contrast, the conduction electronic density in the vdWs gap is always low for all the Cu‐doped, I‐doped, and undoped cases. It indicates that the conduction electrons are spatially separated from the intercalated Cu atoms, while significantly overlapped with the I atoms, especially those substitute Te(2). As a result, the electronic scattering caused by the intercalated Cu atoms is selectively suppressed, leading to higher electron mobility for the thermal‐driven Cu‐intercalated specimens than the CuI‐doped specimens as discussed previously. Such a high‐mobility mechanism is generally called self‐modulation doping because it happens spontaneously in a single‐phase material without requiring a heterojunction, as schematically illustrated in Figure 1a, and was also observed in layered semiconductor Bi_2_O_2_Se.^[^ ^42^ ^]^

**Figure 6:**
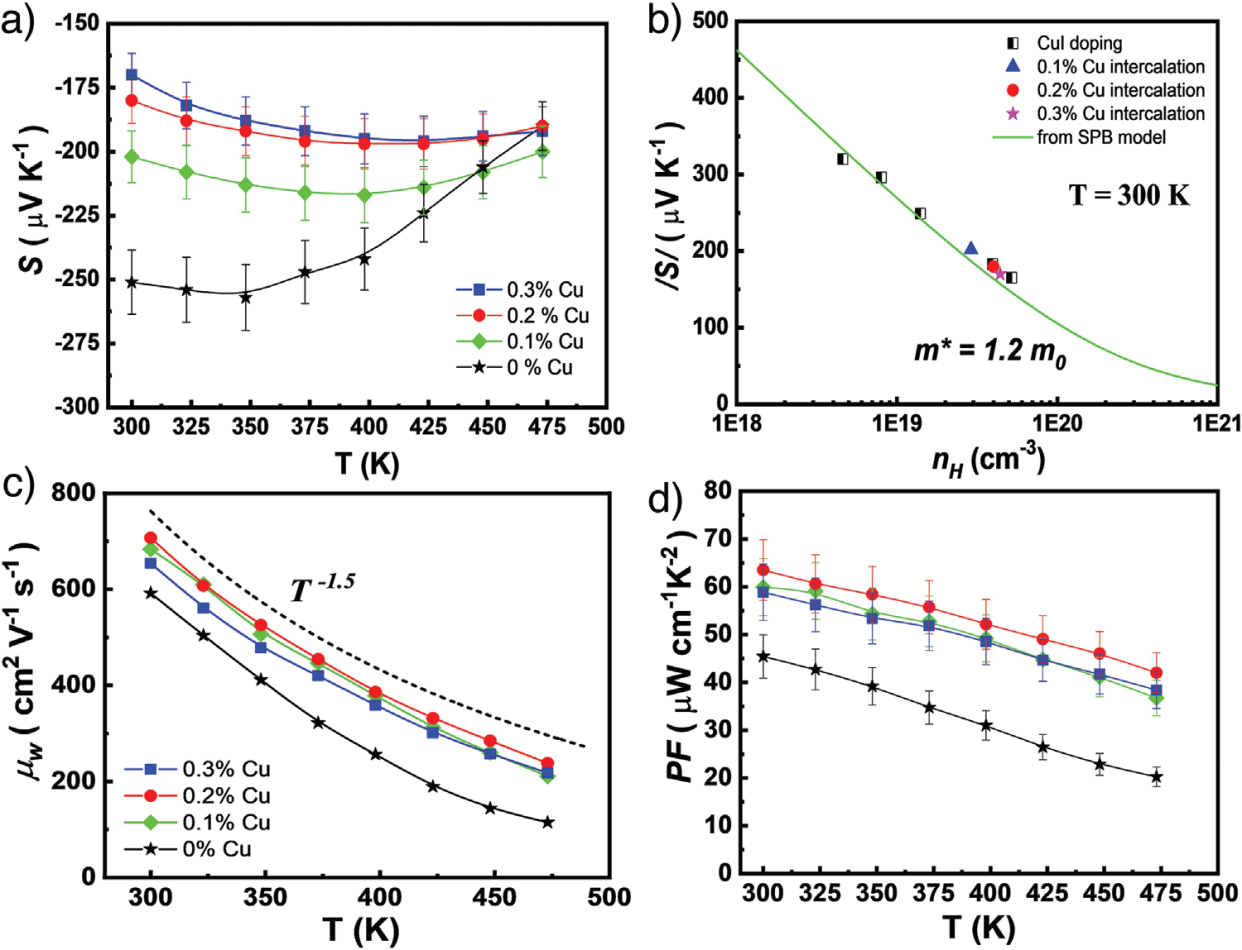
Temperature dependence of TE properties of (CuI)_0.002_Bi_2_Te_2.7_Se_0.3_ + *y* % Cu crystals. a) Seebeck coefficients. b) Room‐temperature Pisarenko relationship with effective mass, m* = 1.2 *m_e_*. c) Weighted mobility. d) PK.

All samples of Cu intercalation show n‐type conduction and the variation of the Seebeck coefficient is roughly similar to the behavior caused by CuI doping (**Figure** **6**a). The decreased Seebeck coefficient is mainly attributed to the increased *n*_H_. Since Bi_2_Te_2.7_Se_0.3_ is a semiconductor with a narrow bandgap, the bipolar effect becomes significant at elevated temperatures and may cause a diminished Seebeck coefficient in the high‐temperature region. We plot the relationship between *S* and *n*_H_ (Pisarenko relation) at 300K based on an SPB model with an acoustic phonon scattering assumption (Figure 6b). The experimental data are well fitted to the calculated curve of the effective mass m* = 1.2 *m_e_*. The analysis suggests that Cu intercalation in the Bi_2_Te_2.7_Se_0.3_ alloys scarcely affects the band structure near the Fermi level. Figure 6c shows the temperature dependence of weighted mobility (*μ_w_*) of all Cu intercalation samples, which is calculated using the measured Seebeck coefficient and electrical resistivity. It reflects intrinsic charge transport properties.^[^ ^43^ ^]^ Obviously, the *μ_w_* of all samples follows a T^−1.5^ dependence, indicating that carriers are predominantly scattered by the acoustic phonons. Cu intercalation indeed enhances the *μ_w_*, and a higher *μ_w_* usually characters a higher PF. Figure 6d presents the temperature dependence of the power factor (PF = *S* ^2^ *σ*). Because of the significantly enhanced *σ* in Cu intercalated samples, the corresponding PFs are substantially improved in the entire measurement temperatures. An ultrahigh PF of ≈63.5 µW cm^−1^ K^−2^ at 300 K is achieved for the sample of (CuI)_0.002_Bi_2_Te_2.7_Se_0.3_ + 0.2% intercalated Cu, which is ≈1.4 times larger than that of pristine (CuI)_0.002_Bi_2_Te_2.7_Se_0.3_. To our best knowledge, the achieved maximum PF is probably the highest record to date among single‐crystalline and polycrystalline *n*‐type Bi_2_Te_3_‐based materials.^[^ ^44^ ^]^ However, a continued increase in the Cu content does not further enhance PF owing to the deterioration of Seebeck coefficient near room temperature and degradation of electrical conductivity at higher temperatures. An appropriate amount of 0.2% Cu intercalation is required to maximize ZT.

### 2.3 Suppression of Lattice Thermal Conductivity

**Figure 7:**
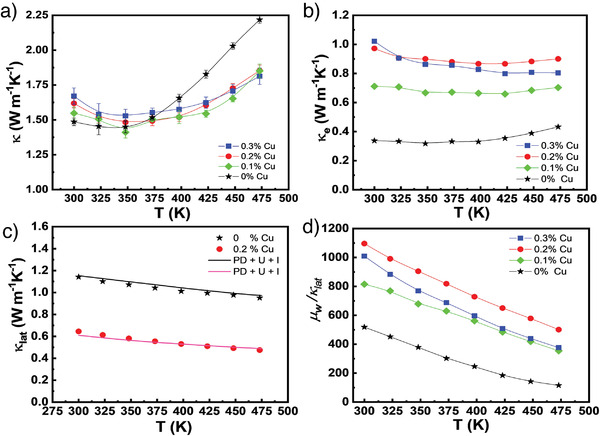
Temperature dependence of thermal properties of (CuI)_0.002_Bi_2_Te_2.7_Se_0.3_ + *y* % Cu crystals. a) Total thermal conductivity. b) Electrical thermal conductivity. c) Lattice thermal conductivity. The solid lines show the fitting curves using the Debye–Callaway model. d) The ratio of weighted carrier mobility to lattice thermal conductivity (*μ_w_*/ *κ*_lat_) as a function of temperature.

The total thermal conductivity (*κ*) as a function of temperature for the (CuI)_0.002_Bi_2_Te_2.7_Se_0.3_ + *y* % intercalated Cu (*y* = ≈0–0.3) is shown in **Figure** **7**a. Cu intercalation increases the *κ* near 300 K. Generally, *κ* is the sum of the lattice contribution (*κ*_lat_) and electronic contribution (*κ*_ele_). *κ*_ele_ is proportional to the *σ* and can be calculated by the Wiedemann–Franz law, *κ*_ele_ = *Lσ*T, where *L* is the Lorenz number. The values of *L* for all samples here were estimated using an SPB model.^[^ ^45^ ^]^ As the temperature increases, in addition to *κ*_ele_ and *κ*_lat_, bipolar thermal conductivity (*κ*_b_) starts to contribute to *κ*. Here we examine their portion to *κ* and reveal the influence of Cu intercalation on the thermal transport of materials. The *κ*_ele_ raises as the concentration of intercalated Cu atoms increases (Figure 7b). The calculation of *κ*_b_ is very challenging because it needs to consider many band structure parameters, such as effective mass, mobility, and bandgap.^[^ ^46^ ^]^ Here we use an alternative method to calculate *κ*_b_ indirectly,^[^ ^6^ ^]^ and then obtain *κ*_lat_. As we know, at temperatures close to 300 K or lower, the bipolar effect is probably negligible, so *κ* − *κ*_ele_ can be used to assess *κ*_lat_ more reliably. Furthermore, the temperature region that we are discussing is higher than the Debye temperature (Θ_D_ ≈164 K for Bi_2_Te_2.7_Se_0.3_),^[^ ^47^ ^]^ so the Umklapp process dominates the phonon propagation. *κ*_lat_ follows the 1/T temperature dependence. As a result, the formula *κ*_lat_ = AT^−1^ + B (where A and B are fitting factors) was applied to fit the *κ* − *κ*_ele_ data in an appropriate temperature range. We obtain a function of *κ*_lat_ and use it to calculate the *κ*_lat_ at higher temperatures. The detailed fitting parameters and *κ*_b_ for all samples are summarized in Figure S6, Supporting Information. Interestingly, the samples with Cu intercalation have lower *κ*_b_ in the high‐temperature region, which is beneficial for the ZT enhancement at elevated temperatures. The significant reduction in *κ*_b_ may be roughly understood as the fact that when a large number of Cu atoms are intercalated in the vdWs gaps, the created nanoscale interfaces may preferentially scatter these minority carriers.^[^ ^48^, ^49^ ^]^

Figure 7c plots the variation of estimated *κ*_lat_. The *κ*_lat_ values of the Cu‐intercalated specimen are substantially lower than that of the non‐intercalated one. *κ*_lat_ basically decreases with increasing Cu content. At 375 K, the *κ*_lat_ of the non‐intercalated specimen is 1.06 Wm^−1^K^−1^, whereas it is only 0.55 Wm^−1^K^−1^ for 0.2% Cu intercalated specimen, showing a 48% reduction in *κ*_lat_. Obviously, the intercalation of foreign Cu atoms can account for most of the change in *κ*. To quantitatively understand the role of phonon scattering, the *κ*_lat_ values of the specimens were calculated using the Debye–Callaway model,^[^ ^50^ ^]^ as given by Equation (1).

(1) $$\kappa_{\mathrm{lat}}=\frac{k_{B}}{2\pi^{2}\upsilon }\;{\left(\frac{k_{B}T}{\hbar }\right)}^{3}\int_{0}^{\theta_{D}/T}\tau_{c}\frac{x^{4}e^{x}}{{\left(e^{x}-1\right)}^{2}}dx$$

where x = ħ*ω/k*_B_ *T* is the reduced phonon frequency, *ω* is the phonon frequency, *υ* is the phonon velocity, *θ*_D_ is the Debye temperature, ℏ is the reduced Planck constant, *k*_B_ is the Boltzmann constant, and the total relaxation time *τ_c_* is a reciprocal sum of the relaxation times of the relevant phonon scattering mechanisms. The *τ_c_* was estimated by taking account of the point defects (*τ*_PD_), Umklapp (*τ*_U_) process, planar defects (*τ*_I_), and calculated according to the Matthiessen's rule, as given by Equation (2).

(2) $$\frac{1}{\tau_{c}}=\;A\omega^{4}+\left(B_{U}Te^{-\frac{\theta_{D}}{3T}}+\;B_{I}\right)\omega^{2}\;$$

where *A* is the point defect scattering parameter, *B*_U_ is the parameter for Umklaap scattering, and *B*_I_ is correlated to the phonon scattering of interface or planar defects.^[^ ^51^ ^]^ The inclusion of *B*_I_ substantially improves the fitting quality of *κ*_lat_ for the specimen with Cu intercalation in the 300–475 K temperature range. **Table** **2** shows the fitting parameters. The scattering parameters A and *B*_U_ do not show a discernible trend, but *B*_I_ presents a significant difference. For the specimen with 0.2% Cu intercalation, the best fitting for *κ*_lat_ was obtained when the density of interface or planar defects ≈10^8^ m^−1^, which corresponds to *B*_I_ ≈ 6×10^−15^ s. The fitted *B*_I_ is about 100 times larger than the value for the non‐intercalated one, suggesting a stronger defect scattering like stacking faults in Cu intercalated specimen. Since the specimen with Cu intercalation may form the Cu/Bi_2_Te_2.7_Se_0.3_ pseudo‐superlattice structure, it is expected to have such a high density of interface or planar defects. Therefore, the reduction in *κ*_lat_ for the intercalated specimen is mainly attributed to the interface/planar defect scattering induced by Cu intercalation. We also calculated the value of *μ_w_*/*κ*_lat_, which is proportional to the TE quality factor,^[^ ^43^ ^]^ to comprehensively evaluate the role of Cu intercalation in (CuI)_0.002_Bi_2_Te_2.7_Se_0.3_ crystals (Figure 7d). Cu intercalation really raises the value of *μ_w_*/*κ*_lat_ over the entire temperature, and a significant enhancement of at least 120% is achieved in the sample with 0.2% Cu intercalation. The extraordinarily high value of *μ_w_*/*κ*_lat_ demonstrates that the Cu intercalation in (CuI)_0.002_Bi_2_Te_2.7_Se_0.3_ can effectively scatter phonons while preserving good electrical transport properties, exhibiting a phonon glass and electronic crystal behavior.^[^ ^52^ ^]^

**Table 2:** Fitting parameters used in the Debye–Callaway mode, where *A* is the point defect scattering parameter, *B*_U_ is the parameter for Umklaap scattering, and *B*_I_ is correlated to the phonon scattering of interface or planar defects, respectively

Scattering parameters	(CuI)_0.002_Bi_2_ Te_2.7_Se_0.3_	(CuI)_0.002_Bi_2_Te_2.7_Se_0.3_ + 0.2% Cu intercalated
	Fitted (solid black, Figure 7c)	Fitted (solid red, Figure 7c)
*A* (s^3^)	7.4 × 10^−41^	7.4 × 10^−41^
*B*_U_ (sK^−1^)	1.2 × 10^−17^	1.2 × 10^−17^
*B*_I_ (s)	8.0 × 10^−17^	6.0 × 10^−15^

**Figure 8:**
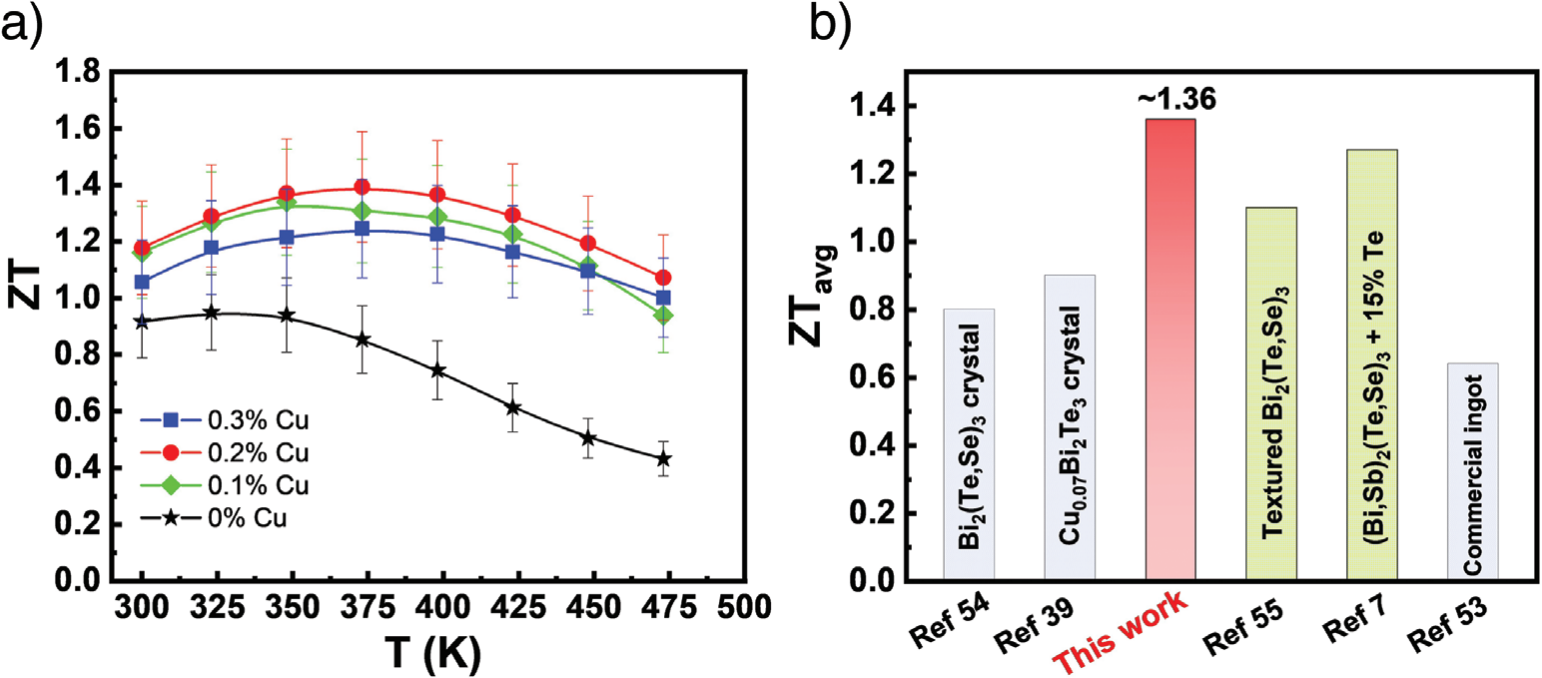
TE performance of (CuI)_0.002_Bi_2_Te_2.7_Se_0.3_ + *y* % Cu crystals. a) ZT values. b) Average ZT values for advanced *n*‐type Bi_2_Te_3_‐based single crystals and textured alloys.

**Figure** **8**a shows the temperature dependences of ZT for all (CuI)_0.002_Bi_2_Te_2.7_Se_0.3_ + y % Cu specimens. All Cu‐intercalated specimens show a remarkable enhancement in ZT values over the entire temperature. Especially, the sample of (CuI)_0.002_Bi_2_Te_2.7_Se_0.3_ + 0.2% Cu reaches a maximum ZT of 1.42 at 375 K, showing an approximately 75% enhancement over the pristine (CuI)_0.002_Bi_2_Te_2.7_Se_0.3_ sample. As can be seen, the synergistic modulation of PF and thermal conductivity by Cu intercalation in the vdW gap of layered materials opens a new avenue for achieving high ZT in the *n*‐type Bi_2_Te_2.7_Se_0.3_ materials. The result is comparable to that published by Zhu et al. recently,^[^ ^7^ ^]^ but the method and mechanism for enhancing the ZT are completely different. Furthermore, the sample of (CuI)_0.002_Bi_2_Te_2.7_Se_0.3_ + 0.2% Cu also shows an average ZT (ZT_avg_) of 1.36 ranging from 300 to 450 K (Figure 8b), which is about 100% higher than that of commercial materials (ZT_avg_ ≈ 0.64) and among the best‐reported record for Bi_2_Te_3_‐based single crystals and textured alloys.^[^ ^39^, ^53^, ^54^, ^55^ ^]^ We also examined the reproducibility of the Cu intercalation fabrication process from several batches of 0.2% Cu samples by cycling TE performance, and confirmed the excellent repeatability and thermal stability of the Cu‐intercalated samples (Figures S7 and S8, Supporting Information).

## 3 Conclusion

This work presents an innovative strategy to synthesize n‐type Bi_2_Te_2.7_Se_0.3_ crystals with exceptional TE performance via thermal‐driven Cu intercalation in the vdW gaps. The intercalated Cu atoms act as modulation doping and contribute conduction electrons of wavefunction spatially separated from the Cu atoms themselves, which improves carrier mobility while maintaining the carrier concentration similar to that in the uniformly doped sample. Meanwhile, the Cu/Bi_2_Te_2.7_Se_0.3_ pseudo‐superlattice structure formed by Cu atoms intercalated into the vdW gaps is very beneficial to enhancing phonon scattering, thereby reducing the lattice thermal conductivity. The significant enhancement of PF and the reduced thermal conductivity collectively achieve a record high ZT of 1.42 at 375 K and an average ZT of 1.36 from 300 to 450 K in (CuI)_0.002_Bi_2_Te_2.7_Se_0.3_ + 0.2% Cu sample. Our work suggests that the intercalation of Cu atoms within the vdWs gaps in the layered structure is an effective approach to modulating phonon and carrier transport in n‐type bismuth telluride‐based materials.

## 4 Experimental Section

### Synthesis

The crystal with nominal composition (CuI)_x_Bi_2_Te_2.7_Se_0.3_ (x = 0, 0.001, 0.002, 0.003, and 0.004) were prepared using the Bridgman method. High‐purity elements of Bi (99.999%, Alfa Aesar), Te (99.999%, Alfa Aesar), Se (99.999%, Alfa Aesar), and CuI (99.998%, Alfa Aesar) were weighted according to the stoichiometric ratio. The sealed silica tube was heated at 1123 K for 24 h in order to homogenize the molten liquid, cooled to 1023 K, and held at temperature for 12 h. The tube was pulled down at 1023 K along with a growth rate of 5 mm h^−1^. The grown single crystals were approximately 50–60 mm long, 13 mm in diameter, and well cleavable (Figure S9, Supporting Information). The cleavage plane of crystals was parallel to the pulling direction. For the intercalation of Cu atoms, the (CuI)_0.002_Bi_2_Te_2.7_Se_0.3_ crystal with excellent TE properties was selected as the base material for subsequent copper intercalation studies. Three different thickness Cu films were then thermally evaporated on the surface that was perpendicular to the basal plane of the (CuI)_0.002_Bi_2_Te_2.7_Se_0.3_crystal and annealed at ≈670–800 K for several days. The number of intercalated Cu atoms was thus calculated by the film thickness. A series of (CuI)_0.002_Bi_2_Te_2.7_Se_0.3_ + *y* mol. % Cu (*y* = 0.1, 0.2, and 0.3) crystals were prepared with the same fabrication procedure.

### Characterization

The structural phase of crystals was analyzed by X‐ray diffraction, carried out with a diffractometer (XRD, PANalytical X'Pert Pro) equipped with Cu K*α* radiation (*λ* = 1.5406 Å). The Rietveld refinement was performed to determine the lattice parameters of crystals. Furthermore, the crystal orientations of samples were analyzed using a triple‐axis X‐ray diffractometer (Malvern Panalytical's Materials Research Diffractometers, MRD) and a Laue diffractometer (IPX‐YGR‐LC). The microstructures and elemental composition analysis of the crystals were examined using scanning electron microscopy (SEM, Inspect F FEI) equipped with energy‐dispersive X‐ray spectroscopy (EDX). The compositions of the phase and the distribution of elements were identified by an electron probe microanalyzer (EPMA JXA‐8200, JEOL). The crystal with ≈50–80 nm in thickness was prepared for the transmission electron micrographs (TEM) observation using a Focused Ion Beam instrument (Hitachi NX2000). The high‐resolution TEM imaging was conducted using a spherical aberration‐corrected transmission electron microscope (JEOL‐ARM 200FTH) operating at 200 kV. Raman spectra of the specimens at room temperature were measured by a Raman microscope (Horiba Jobin Yvon) which employs a continuous‐wave 532 nm laser to excite the sample and detect the Raman scattering and shifts of vibrational frequencies with a spectral resolution of ≈2 cm^−1^. The X‐ray photoelectron spectroscopic analysis (XPS, JEOL, JAMP‐9500F) was performed to investigate the valence of Cu in the crystals.

### Thermoelectric Property Measurement

The electrical resistivity and Seebeck coefficient of samples were measured using a commercially available instrument (ZEM‐3, ULVAC‐RIKO, Japan) under a helium atmosphere from 300 to 475 K. The uncertainty for the electrical conductivity was 3%, and the Seebeck coefficient was 5%. The thermal conductivity of the bulk sample was calculated from the relationship: *κ* = *λρC_p_*, where *λ*, *ρ*, *C_p_* are thermal diffusivity, mass density, and specific heat, respectively. Mass density was measured by the Archimedes method. Specific heat was calculated according to the Dulong–Petit Law, and the thermal diffusivity was measured by a laser flash apparatus (LFA‐457, NETZSCH). The uncertainty of the thermal conductivity was 5%. Combining several uncertainties from Seebeck coefficient, electrical conductivity, and thermal conductivity, the total uncertainty of ZT was about 18%. All transport properties were measured along the same direction. The carrier concentration of the sample was calculated using *n*_H_  =   − 1/*eR*_H_, where *R*_H_ is the Hall coefficient measured by a commercial Quantum Design Physical Property Measurement System (PPMS, Quantum Design) via scanning a magnetic field from −2T to +2T. The uncertainty of the Hall coefficient was ≈3%.

### DFT Calculation

The electronic structure and the partial‐charge density of electronic states were calculated through the projector augmented wave (PAW) approach within the framework of density functional theory as implemented in the Vienna ab initio Simulation Package (VASP).^[^ ^56^, ^57^, ^58^ ^]^ The exchange‐correlation was described in the Perdew–Burke–Ernzerhof (PBE) form of generalized gradient approximation (GGA).^[^ ^59^, ^60^ ^]^ The vdWs interactions are based on the DFT‐D3 method with Becke–Jonson damping.^[^ ^61^, ^62^ ^]^ A 9 × 9 × 2 Γ centered Monkhorst–Pack k‐mesh was used for the integration of Brillouin zone.^[^ ^63^ ^]^ The cutoff energy for the plane‐wave basis was set as 400 eV. All the internal atomic coordinates and the lattice constant were relaxed until the magnitude of the force acting on all atoms was less than 0.02 eV Å^−1^ and the total energy converges within 10^−8^ eV.

## Conflict of Interest

The authors declare no conflict of interest.

## Supporting information

Supporting information

## Data Availability

The data that support the findings of this study are available from the corresponding author upon reasonable request.
