# Supplementary material for: Modulation Doping Enables Ultrahigh Power Factor and Thermoelectric ZT in n‐Type Bi2Te2.7Se0.3
Source: Adv Sci (Weinh). 2022 Apr 27;9(20):2201353. doi: 10.1002/advs.202201353 (PMC9284191; doi:10.1002/advs.202201353)
Supplement: Supplementary file 1 — Supporting information [file ADVS-9-2201353-s001.pdf]

Supporting Information

Modulation doping enables ultrahigh power factor and thermoelectric ZT in n-type $Bi_2Te_{2.7}Se_{0.3}$

Cheng-Lung Chen,[1,#] Te-Hsien Wang,[2] Zih-Gin Yu,[1,3] Yohanes Hutabalian,[1] Ranganayakulu K.Vankayala,[1] Chao-Chih Chen,[4] Wen-Pin Hsieh,[4] Horng-Tay Jeng,[5] , Da-Hua Wei,[3] Yang-Yuan Chen[1,6,*]

[1]Institute of Physics, Academia Sinica, Taipei 11529, Taiwan, ROC
[2]Department of Physics, National Chung Hsing University, Taichung 40227, Taiwan, ROC
[3]Graduate Institute of Manufacturing Technology, National Taipei University of Technology, Taipei 10608, Taiwan, ROC
[4]Institute of Earth Sciences, Academia Sinica, Taipei 11529, Taiwan, ROC
[5]Department of Physics, National Tsing Hua University, Hsinchu 30013, Taiwan, ROC
[6]Graduate Institute of Applied Physics, National Chengchi University, Taipei 11605, Taiwan, ROC

# Corresponding author: clchen0417@gmail.com
*Corresponding author: cheny2@phys.sinica.edu.tw

1. Structural characterization in $(CuI)_xBi_2Te_{2.7}Se_{0.3}$ (x=0 ~ 0.004) crystals

The powder x-ray diffraction patterns of CuI doping samples are shown in Figure S1a. All the patterns are indexed to the rhombohedral lattice structure of Bi_2Te_3 with the space group of R3m with slight peak shifts that caused by CuI doping. The lattice constants as a function of dopant content is shown in Figure S1b. For all the specimens in the series, the change in lattice parameter *a* and *c* is not discernible. It is known that I^- and Te^{2-} have almost identical ionic radii, and the change in lattice parameters caused by the substitution of I for Te is negligible. As for Cu atoms, in addition to entering the van der Waals gap and occupying the interstitial site between the two quintet. They may also form copper nano-precipitates and disperse in the matrix. From the TEM images of $(CuI)_{0.003}Bi_2Te_{2.7}Se_{0.3}$ (Figure S1c), we observed several nano-precipitation of approximately 3–7 nm along the *c*-axis. More importantly, these Cu-rich nanoscale precipitates show a semi-coherent boundary between the matrix and precipitate. Figure S2a shows the Raman spectra of $(CuI)_xBi_2Te_{2.7}Se_{0.3}$ (x=0 ~ 0.004) crystals. Two prominent peaks at 103 cm^{-1} and 136 cm^{-1} are assigned to E_g^2 and A_{1g}^2 modes, respectively. Theoretically, the A_{1g}^2 mode corresponds to the vibration mode along the *c*-axis; the E_g^2 mode corresponds to the in-plane vibrations that are perpendicular to the c-axis. Obviously, $(CuI)_xBi_2Te_{2.7}Se_{0.3}$ (x=0.001 ~ 0.004) samples show very similar Raman active phonon modes (A_{1g}^2, E_g^2) like undoped $Bi_2Te_{2.7}Se_{0.3}$ sample. This means that the doped Cu atoms are probably located in the van der Waals gap of $Bi_2Te_{2.7}Se_{0.3}$ rather substituting Bi cations from the lattice. Figure S2b shows the x-ray photoelectron spectrum (XPS) of $(CuI)_{0.002}Bi_2Te_{2.7}Se_{0.3}$ crystals. The characteristic peaks for zero-valence Cu of sample are observed at 933 and 953 eV for Cu $2p_{3/2}$ and Cu $2p_{1/2}$, respectively. Since no satellite peaks between two main peaks were detected, it can definitely demonstrate that the presence of zero-valence Cu in $(CuI)_xBi_2Te_{2.7}Se_{0.3}$.

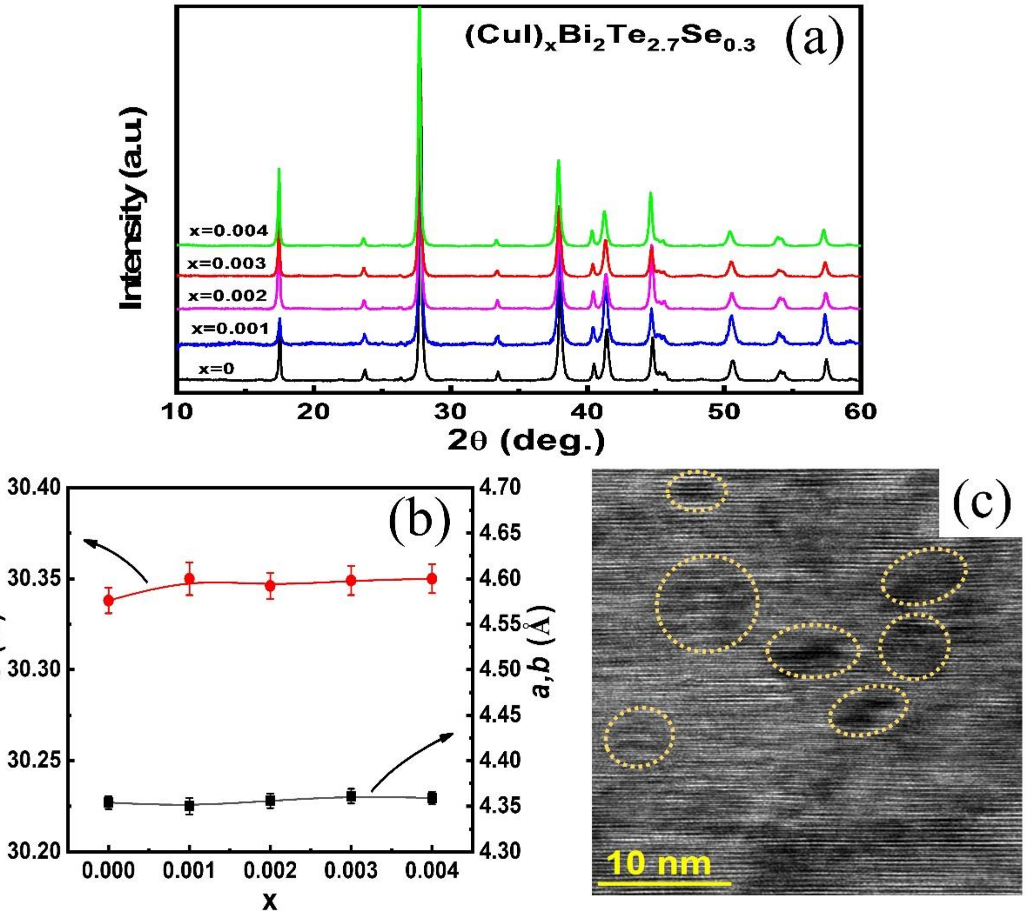

Figure S1: Structural characterization of $(CuI)_xBi_2Te_{2.7}Se_{0.3}$ (x=0 ~ 0.004) crystals. (a) Comparison of XRD patterns. (b) Variation of lattice constants of $(CuI)_xBi_2Te_{2.7}Se_{0.3}$ crystals. (c) HRTEM images showing the nano-precipitation size of about 3–7 nm along the [001] zone axis in $(CuI)_{0.003}Bi_2Te_{2.7}Se_{0.3}$.

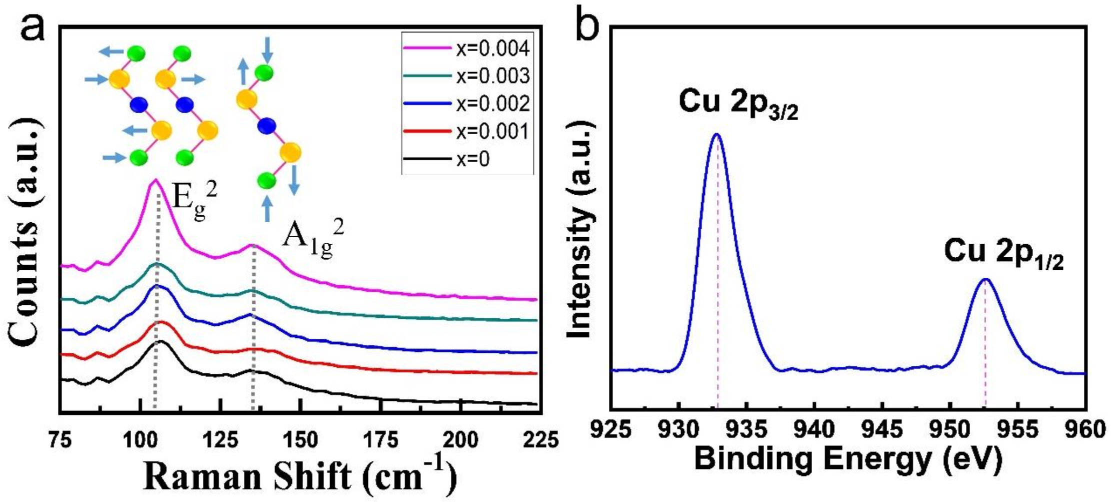

Figure S2: Characterization of $(CuI)_xBi_2Te_{2.7}Se_{0.3}$ (x=0 ~ 0.004) crystals. (a) Comparison of the Raman spectra for all samples. (b) The selected XPS spectra nearby the Cu 2*p* orbitals for $(CuI)_{0.002}Bi_2Te_{2.7}Se_{0.3}$ crystal presenting only two Cu zero-valence characteristic peaks at 933 and 953 eV for Cu $2p_{3/2}$ and Cu $2p_{1/2}$, respectively.

**Nominal composition** $(CuI)_xBi_2Te_{2.7}Se_{0.3}$	**Carrier type**	**σ (S cm^{-1})**	n_H **(×10^{19} cm^{-3})**	μ **(cm^2 V^{-1} s^{-1})**	S **(μV K^{-1})**
**x = 0.00**	***p***	**211**	**0.4**	**330**	**+320**
**x = 0.001**	***n***	**313**	**0.8**	**244**	**-296**
**x = 0.002**	***n***	**721**	**1.4**	**321**	**-251**
**x = 0.003**	***n***	**1240**	**3.9**	**198**	**-183**
**x = 0.004**	***n***	**1343**	**5.2**	**161**	**-165**

Table S1: The Hall carrier concentration (n_H), mobility (μ), electrical conductivity (σ), and Seebeck coefficient (S) of $(CuI)_xBi_2Te_{2.7}Se_{0.3}$ (x=0 ~ 0.004) crystals at 300 K.

2. Thermoelectric transport properties of $(CuI)_xBi_2Te_{2.7}Se_{0.3}$ (x=0 ~0.004) crystals

It is well known that the best thermoelectric properties of Bi_2Te_3-based materials are along the basal plane, so all following thermoelectric properties in this work were performed along the basal plane in order to obtain the best thermoelectric transport performance. Figure S3 presents the temperature dependence of thermoelectric

properties for $(CuI)_xBi_2Te_{2.7}Se_{0.3}$ (x=0 ~ 0.004). The electrical conductivity (σ) of the samples gradually increases with the increase of CuI concentration, and changes from a non-degenerate semiconductor to a highly degenerate semiconductor (Figure S3a), which is mainly due to the increase in Hall carrier concentration (n_H). The measured σ, n_H and mobility (μ) for all samples at 300 K are summarized in Table S1. We also quantify the number of free electrons donated by each CuI by analyzing the relationship between Hall carrier concentration and CuI doping concentration. Each CuI can contribute about 1.4 electrons in $(CuI)_xBi_2Te_{2.7}Se_{0.3}$ crystals. Figure S3b shows the temperature-dependent Seebeck coefficients of all samples. The pristine $Bi_2Te_{2.7}Se_{0.3}$ shows a positive Seebeck coefficient (*p*-type) due to the Bi_{Te} anti-site defects. When adding CuI dopants into the $Bi_2Te_{2.7}Se_{0.3}$, the substantial electron donor contributed by Cu- and I-doping will change the Seebeck coefficient to a negative value (*n*-type). With the increase of CuI doping, the room temperature σ significantly enhanced from 313 S cm^{-1} for $(CuI)_{0.001}Bi_2Te_{2.7}Se_{0.3}$ to 1343 S cm^{-1} for the $(CuI)_{0.004}Bi_2Te_{2.7}Se_{0.3}$ along with a decrease of S from -296 μV K^{-1} to -165 μV K^{-1}. The result is very consistent with the increase in carrier concentration (Table S1). We found that the Seebeck coefficient of bismuth telluride is very sensitive to the donor-like defects caused by the cooling rate in preparation process. Five $(CuI)_{0.002}Bi_2Te_{2.7}Se_{0.3}$ crystal samples prepared with the same procedure were examined, and the reproducibility of single crystal preparation was confirmed. CuI doping is essential for accurately controlling the thermoelectric performance of n-type $Bi_2Te_{2.7}Se_{0.3}$ materials during the crystal growth. Figure S3c illustrates the temperature dependence of power factor for all samples. Obviously, CuI doping remarkably improves the power factor of $Bi_2Te_{2.7}Se_{0.3}$ in the entire measurement temperature range. The power factors of CuI doped crystals with x = 0.002, 0.003 and 0.004 peak at 41 μW $cm^{-1}K^{-2}$ around 300 K, and decrease with increasing temperature. The total thermal conductivities (κ) as a function of temperature for all crystals are shown in Figure S3d. The doping of CuI raises κ at 300 K. As the CuI doping level increases from x= 0.001 to 0.004, the corresponding κ also gradually rises from 1.2 to 1.6 W $m^{-1}K^{-1}$ at 300K. Generally, κ is the sum of the lattice contribution (κ_{lat}) and electronic contribution (κ_{ele}). κ_{ele} is proportional to the σ and can be calculated by the Wiedemann–Franz law, $\kappa_{ele} = L\sigma T$, where L is the Lorenz number. The values of L for all samples here were estimated using a single parabolic band (SPB) model with

acoustic phonon scattering. Doping CuI greatly increases the κ_{ele} values. The room temperature κ increments of CuI doped $Bi_2Te_{2.7}Se_{0.3}$ samples mostly come from the contribution of enhanced κ_{ele}. Since $Bi_2Te_{2.7}Se_{0.3}$ belongs to a narrow band gap semiconductor, the contribution of bipolar thermal conductivity (κ_b) to the κ will become more and more obvious as temperature rises (Figure S3e). When the doping content is controlled to x= 0.002~0.003 it has the lowest κ around 325 ~ 400 K. Such reduced κ and enhanced power factor simultaneously result in a significant ZT enhancement in 300 ~ 400 K. As illustrated in Figure S3f, a maximum ZT of 0.6 appears at 300 K for the pristine $Bi_2Te_{2.7}Se_{0.3}$ crystals. With introducing more CuI doping, the maximum ZT enhances and shifts to higher temperatures, i.e., 1.0 at 350K for $(CuI)_{0.002}Bi_2Te_{2.7}Se_{0.3}$ and 1.1 at 373K for $(CuI)_{0.003}Bi_2Te_{2.7}Se_{0.3}$, showing a more than 60 % enhancement over the pristine $Bi_2Te_{2.7}Se_{0.3}$. The significant ZT enhancement is mainly due to the enhanced power factor and reduced thermal conductivity. However, a continued increase in the CuI content does not further enhance ZT owing to the lower Seebck coefficient, mobility and the higher thermal conductivity. An appropriate CuI doping is crucial to maximize ZT by optimizing the thermal and electrical transport properties.

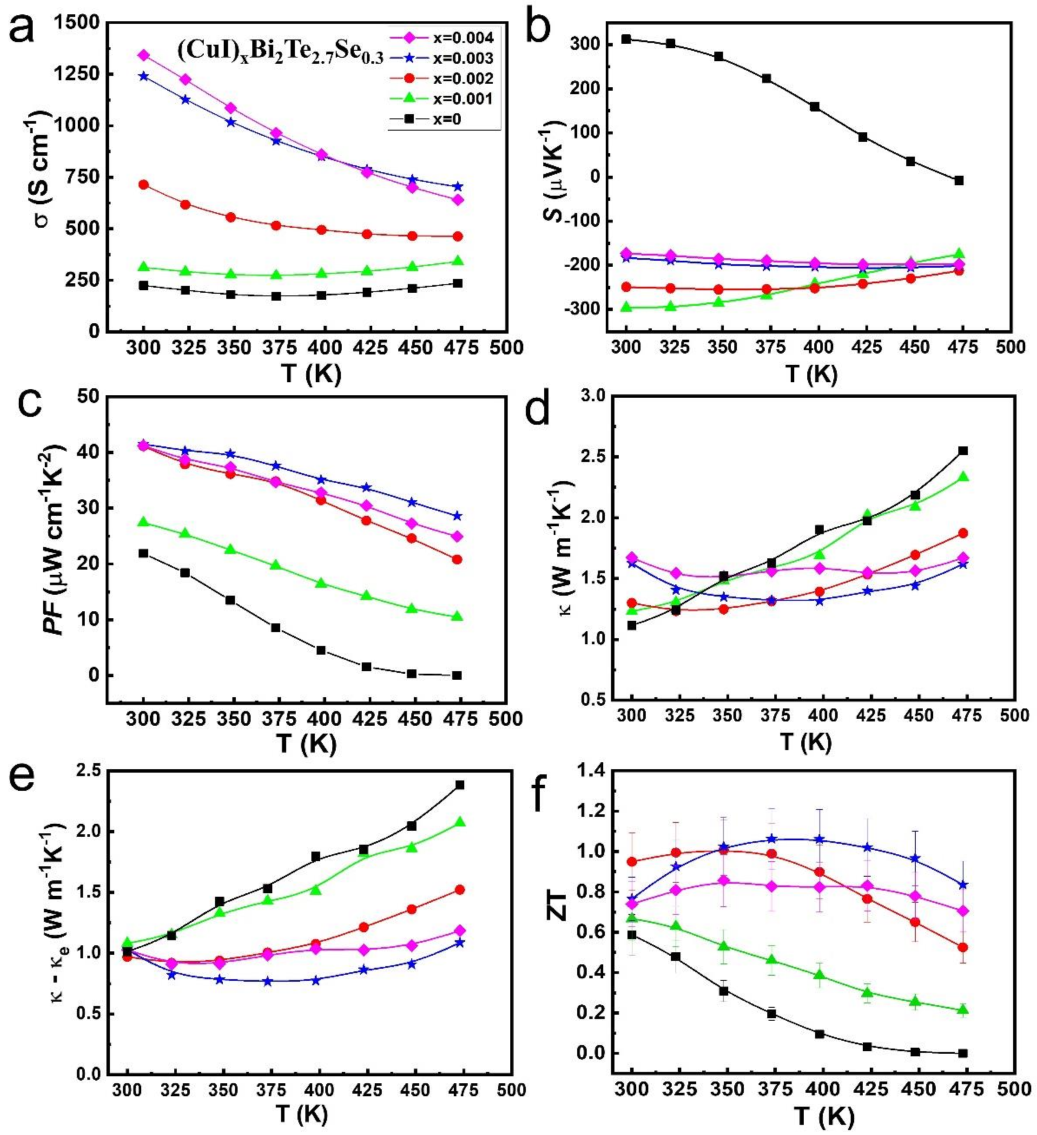

Figure S3: Thermoelectric properties as a function of temperature for $(CuI)_xBi_2Te_{2.7}Se_{0.3}$ (x=0 ~ 0.004) crystals. (a) Electrical conductivity. (b) Seebeck coefficient. (c) Power factor. (d) Total thermal conductivity. (e) The difference of total and electronic thermal conductivity (κ-κ_e). (f) Figure of merit, ZT.

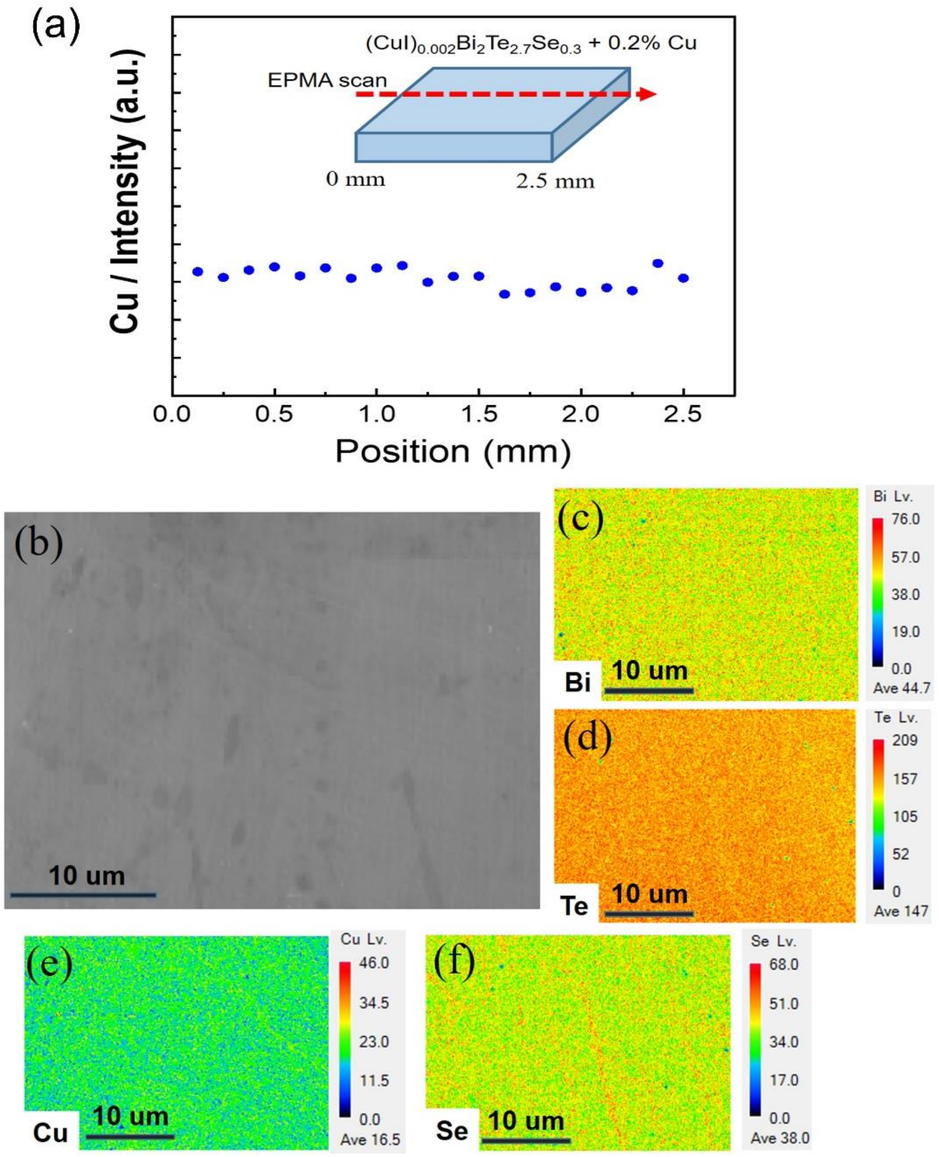

Figure S4: (a) Spatial distribution of Cu element in $(CuI)_{0.002}Bi_2Te_{2.7}Se_{0.3}$ + 0.2 % Cu sample analyzed by the EPMA. (b-f) Microstructures and elemental mapping of $(CuI)_{0.002}Bi_2Te_{2.7}Se_{0.3}$ + 0.2 % Cu sample: (b) Backscattered image, (c) Bi mapping result, (d) Te mapping result, (e) Se mapping result, and (f) Cu mapping result.

3. Charge transfer analysis of Cu-intercalated Bi_2Te_3 crystal structure ($Bi_{24}Te_{36}Cu$)

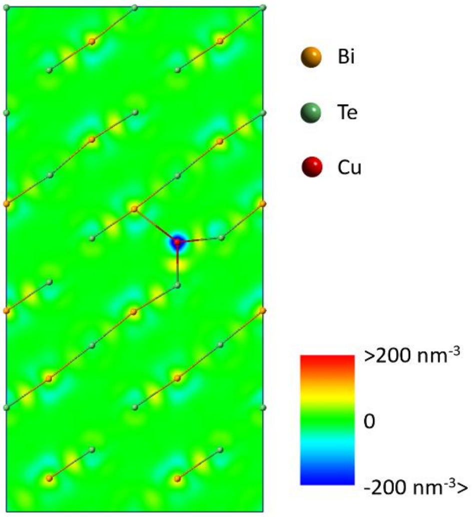

Figure S5: Charge transfer analysis of Cu-intercalated Bi_2Te_3 crystal structure.

4. Inter-valley and Intra-valley scattering

As shown in Figures. 5e-5h, the energy difference between the 2nd CBM and the CBM for doped samples is slightly smaller than that for the undoped sample (Figure. 5h). For the case of a given electron concentration, a smaller energy difference between 2nd CBM and CBM indicates that more portion of electrons lies in the 2nd conduction band valley. This would reduce the intra-valley scattering because fewer electrons lie in the same valley although the inter-valley scattering might be strengthened. The mobility probably can be improved because generally, the inter-valley scattering is much weaker than intra-valley scattering.

5. Change in wave function of conduction elelctrons due to Cu/I impurities

As shown in Figures. 5a-5d, the charge density of the electronic states inside the quintuple layer (QL) closest to the intercalated Cu (Figure. 5a) and that inside the QL whose Te atom is substituted by I (Figures. 5b-c) are higher than that of pure sample (Figure 5d). The reason would be that both I and Cu in our specimens act as donor, and thus they should contribute donor levels around the conduction band minimum (CBM). If we can calculate a unit cell of nearly infinite volume with a single intercalated Cu (or a substituted I) to model the condition of an isolated impurity, we

would see some non-dispersive bands lower than the CBM corresponding to the donor levels and some Cu states merging with Bi_2Te_3 state above the CBM corresponding to the resonant states. In a practical calculation of a finite and even quite small unit cell, which would be more relevant for high doping concentration of our specimens, all the donor (Cu or I) states should merge with the conduction (Bi_2Te_3) states (note that generally the ionization energy of impurities is only about 10 meV). Therefore, the electronic states around the CBM, to some extent, will show the properties of impurity states. i.e., the wave function becomes more localized around the QL which the impurity is closest to as shown in Figures. 5a-5c.

In our experiments, the Cu/I atoms should quite uniformly distribute in each van der Waals gap/QL so that the charge density in each QL should be comparable. The main purpose of Figures. 5a-5d is to show that the density of conduction electrons in the van der Waal gap is low in comparison with that in QL for all the doped and undoped cases we considered.

Reference

[1] Yu, H. J. *et al.* Effects of Cu Addition on Band Gap Energy, Density of State Effective Mass and Charge Transport Properties in Bi_2te_3 Composites. *RSC Adv.* **4**, 43811-43814 (2014).

Fitting parameters for all samples

$\kappa_{lat} = aT^{-1} + b$	$(CuI)_{0.002}Bi_2Te_{2.7}Se_{0.3} + x$ % Cu			
	0 %	0.1 %	0.2 %	0.3 %
a	150.11	199.18	139.42	105.02
b	0.64	0.17	0.18	0.29

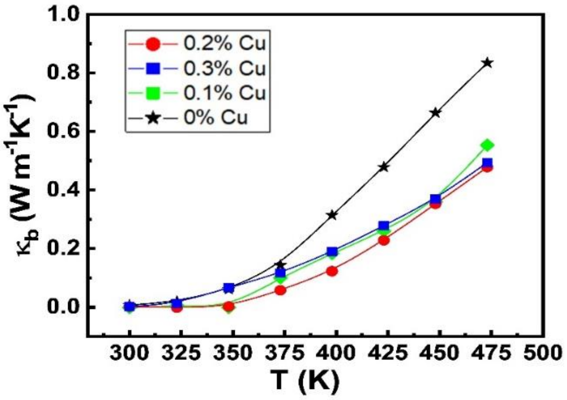

Figure S6: Fitting parameter for $\kappa_{lat} = AT^{-1} + B$, and the bipolar thermal conductivity κ_b for Cu-intercalated samples.

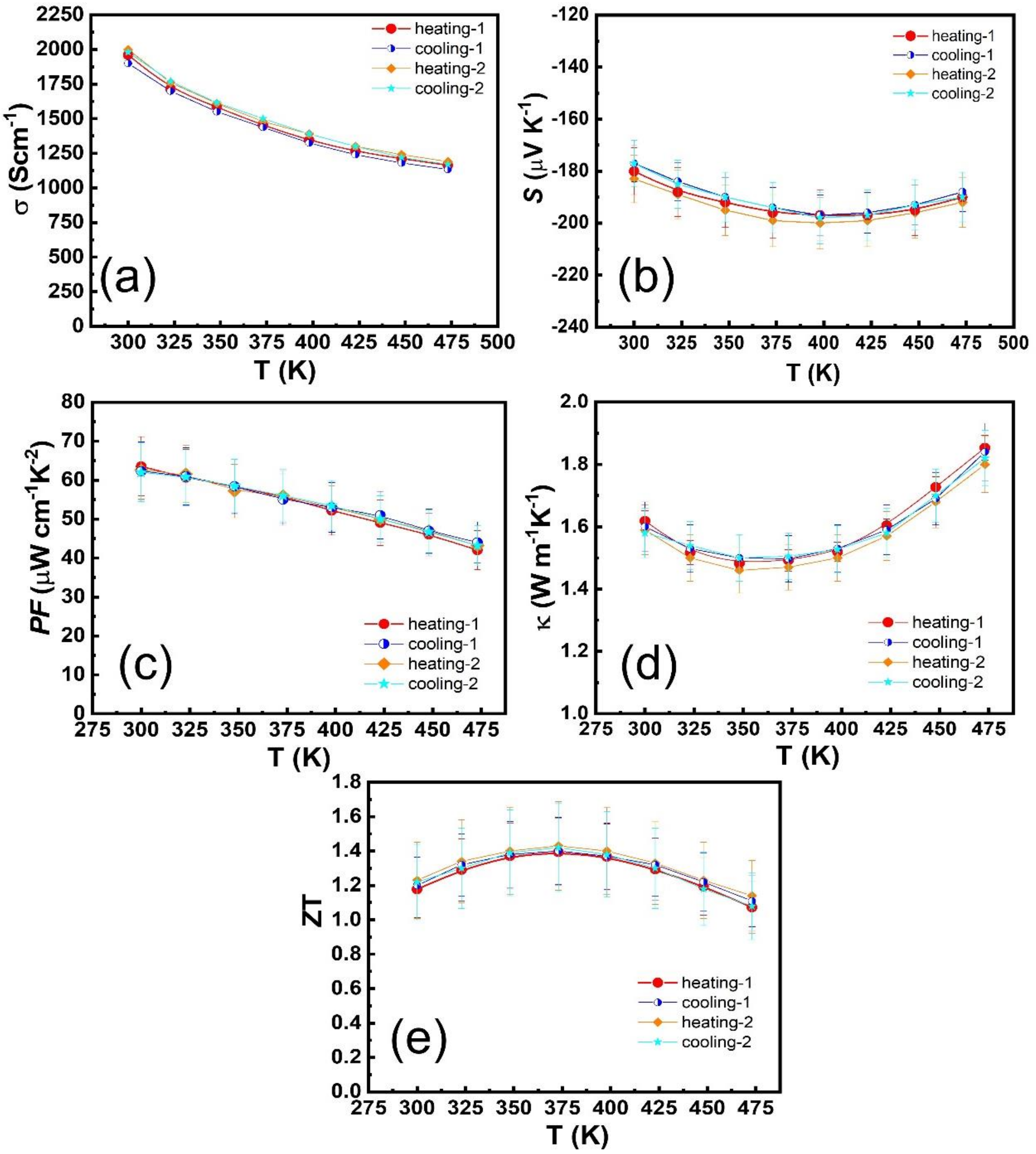

Figure. S7: The heating and cooling measurements for $(CuI)_{0.002}Bi_2Te_{2.7}Se_{0.3}$ + 0.2 % Cu sample, showing a good thermal stability for high ZT value of 1.42 obtained in this work. (a) Electrical conductivity. (b) Seebeck coefficient. (c) Power factor. (d) Thermal conductivity. (e) ZT value.

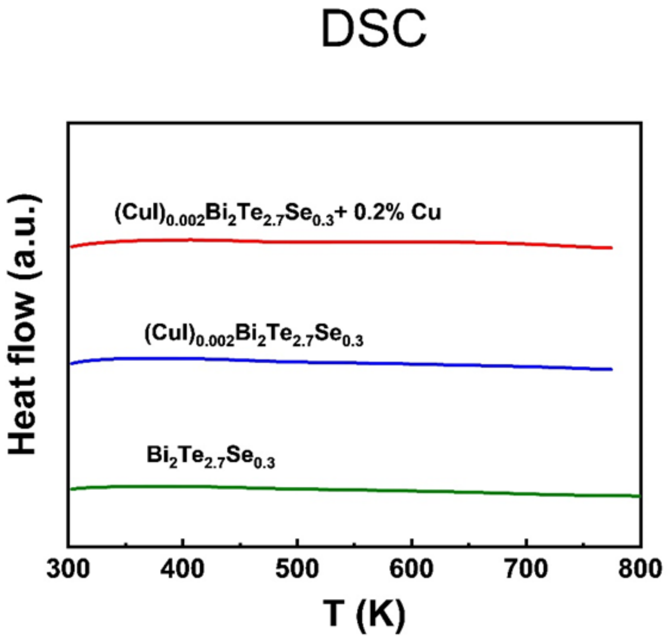

Figure S8: Differential scanning calorimetry (DSC) analysis of, and $(CuI)_{0.002}Bi_2Te_{2.7}Se_{0.3}$ + 0.2% Cu at a rate of 10 K min^{-1} in comparison with the control $Bi_2Te_{2.7}Se_{0.3}$ and $(CuI)_{0.002}Bi_2Te_{2.7}Se_{0.3}$, demonstrating the high thermal stability and phase homogeneity despite the intercalation of Cu atoms.

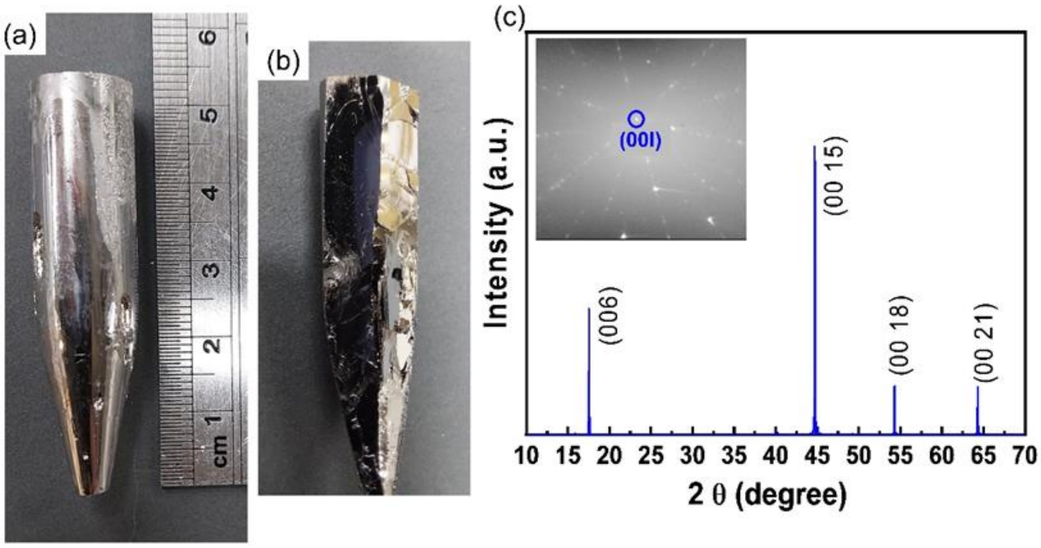

Figure S9: (a) The photograph of a $(CuI)_{0.002}Bi_2Te_{2.7}Se_{0.3}$ single crystal with a length of 53 mm and a diameter of 13 mm grown by the vertical Bridgman furnace method. (b) The crystal with a lustrous cleavage plane and mirror-like surface. (c) X-ray diffraction pattern of $(CuI)_{0.002}Bi_2Te_{2.7}Se_{0.3}$ single crystal. Inset shows the Laue diffraction pattern which reveals the high crystalline quality of the sample. The blue circle near the center of the Laue diffraction pattern represents the <*00l*> planes.
